# C2 Hangman’s fracture treated with posterior cervical fixation with preservation of the semispinalis cervicis muscle: a case report

**DOI:** 10.1097/RC9.0000000000000677

**Published:** 2026-07-08

**Authors:** Kaoru Eguchi, Shunichiro Kuramitsu, Yu Sugiyama, Masashi Ito, Ryo Ando, Satoshi Maesawa

**Affiliations:** Department of Neurosurgery, National Hospital Organization Nagoya Medical Center, Nagoya, Japan

**Keywords:** C2 Hangman’s fracture, cervical rotation, implant removal, posterior cervical fixation, semispinalis cervicis muscle

## Abstract

**Introduction and importance:**

Although posterior fixation is well established for unstable Hangman’s fractures, constructs including C1 often sacrifice cervical rotation, and conventional exposure frequently injures the semispinalis cervicis (SSC). Given the SSC’s essential role in cervical stability, techniques that preserve this muscle may substantially improve postoperative function. However, its preservation during C1–3 fixation has been rarely documented in the literature.

**Case presentation:**

A 30-year-old woman sustained a Levine–Edwards type I Hangman’s fracture from a rollover motor-vehicle accident. Conservative management failed, and CT revealed persistent non-union with an 8-mm fracture gap. Posterior C1–3 fixation with deliberate SSC preservation was performed. The SSC was mobilized laterally without detachment from the C2 spinous process, enabling adequate exposure for screw placement with intraoperative 3D navigation. Postoperative imaging confirmed complete SSC preservation. One year later, after solid bony healing, implant removal was performed. The preserved SSC displayed minimal adhesions, and screw removal proceeded uneventfully. Following removal, the patient experienced significant improvement in cervical rotation and remained asymptomatic.

**Clinical discussion:**

This case demonstrates that SSC preservation is feasible, reproducible, and may meaningfully influence postoperative biomechanics. By minimizing paraspinal muscle damage, this technique may reduce axial symptoms and optimize motion recovery – limitations commonly associated with conventional posterior approaches.

**Conclusion:**

SSC-preserving posterior C1–3 fixation may represent a valuable modification for managing selected Hangman’s fractures, offering potential functional advantages beyond standard techniques. Broader application and long-term evaluation are warranted.

## Introduction

Traumatic spondylolisthesis of the axis (C2), commonly known as a Hangman’s fracture, is a bilateral fracture through the pars interarticularis of C2 and typically results from hyperextension forces. Neurological deficits are uncommon, and patients primarily present with posterior neck pain. The Levine–Edwards classification is widely used to evaluate fracture stability and guide treatment: Type I (<3 mm displacement, no angulation) is generally stable and managed conservatively, whereas Types II, IIa, and III indicate instability and often require surgical fixation.

HIGHLIGHTS

- This case demonstrates posterior C1–3 fixation for an unstable C2 Hangman’s fracture, with preservation of the semispinalis cervicis (SSC) muscle.
- Preservation of the SSC facilitated easier implant removal and maintained cervical rotational function.
- Intraoperative 3D CT navigation enabled precise screw placement, despite limited exposure.
- This technique may minimize muscle-related postoperative pain and functional decline.
- SSC preservation is feasible even in posterior fixation constructs involving C1.

The choice of surgical approach – particularly the degree to which motion segments are preserved – depends on fracture morphology, ligamentous injury, and reduction status. Treatment options include posterior fixation (e.g., C2 pars or pedicle screws), anterior C2–3 discectomy and fusion (ACDF), or combined approaches^[^1–3^]^. Posterior fixation reliably restores anatomic alignment; however, constructs involving C1 can significantly restrict cervical rotation^[^4–7^]^.

The semispinalis cervicis (SSC) muscle originates from the upper thoracic transverse processes and inserts onto the cervical spinous processes, contributing to extension, rotation, and posterior cervical stability. Recent studies on cervical laminoplasty and C2 screw placement have highlighted the importance of SSC preservation in preventing postoperative neck pain and malalignment. Despite these observations, the technical details and clinical implications of SSC preservation in the management of C2 Hangman’s fractures remain poorly described^[^8–11^]^.

Herein, we present a case of a C2 Hangman’s fracture treated with posterior C1–3 fixation and SSC preservation, followed by implant removal. We describe the technical nuances and discuss the potential clinical benefits of SSC preservation for maintaining postoperative cervical function.

This case report has been presented in line with the SCARE checklist^[^12^]^.

## Case presentation

**Figure 1.**
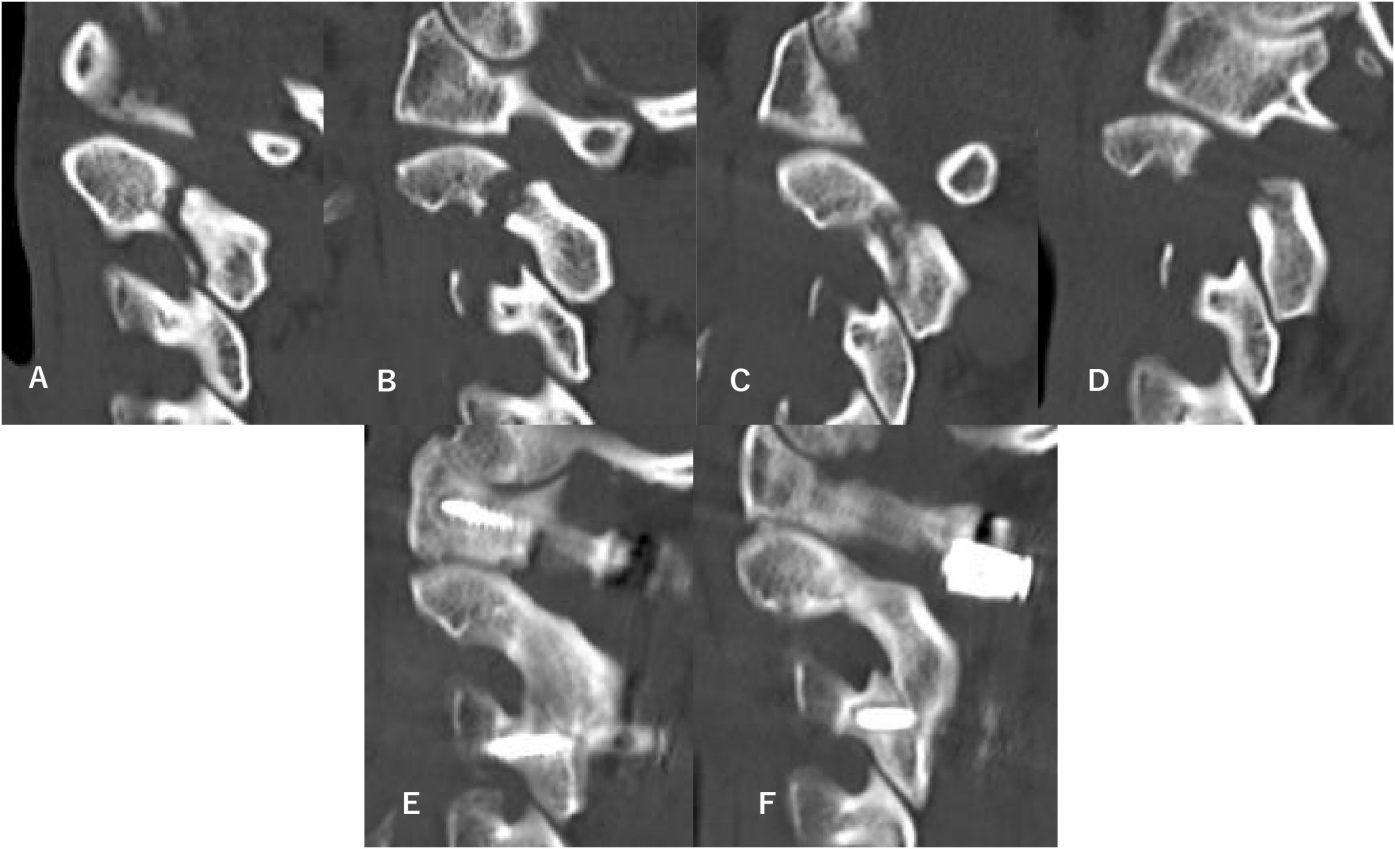
Preoperative and postoperative CT imaging of the C2 Hangman’s fracture. (A and B) Initial CT after injury shows bilateral pars interarticularis fractures with a gap of approximately 8 mm. (C and D) One-month follow-up CT demonstrates nonunion and persistent separation of the fracture site. (E and F) One-year postoperative CT confirms nearly complete bony fusion at the fracture site.

A 30-year-old woman involved in a motor-vehicle rollover accident presented with severe posterior neck pain without neurological deficits. CT demonstrated bilateral pars interarticularis fractures of C2, consistent with a Levine–Edwards type I Hangman’s fracture (Fig. 1A and B). Because the patient declined halo-vest immobilization due to childcare responsibilities, rigid cervical collar immobilization was initiated.

One month later, the patient experienced worsening posterior neck pain. Follow-up CT revealed persistent nonunion with an approximately 8-mm gap at the left pars interarticularis (Fig. 1C and D). Given failed conservative treatment, posterior C1–3 fixation was planned.

**Figure 2.**
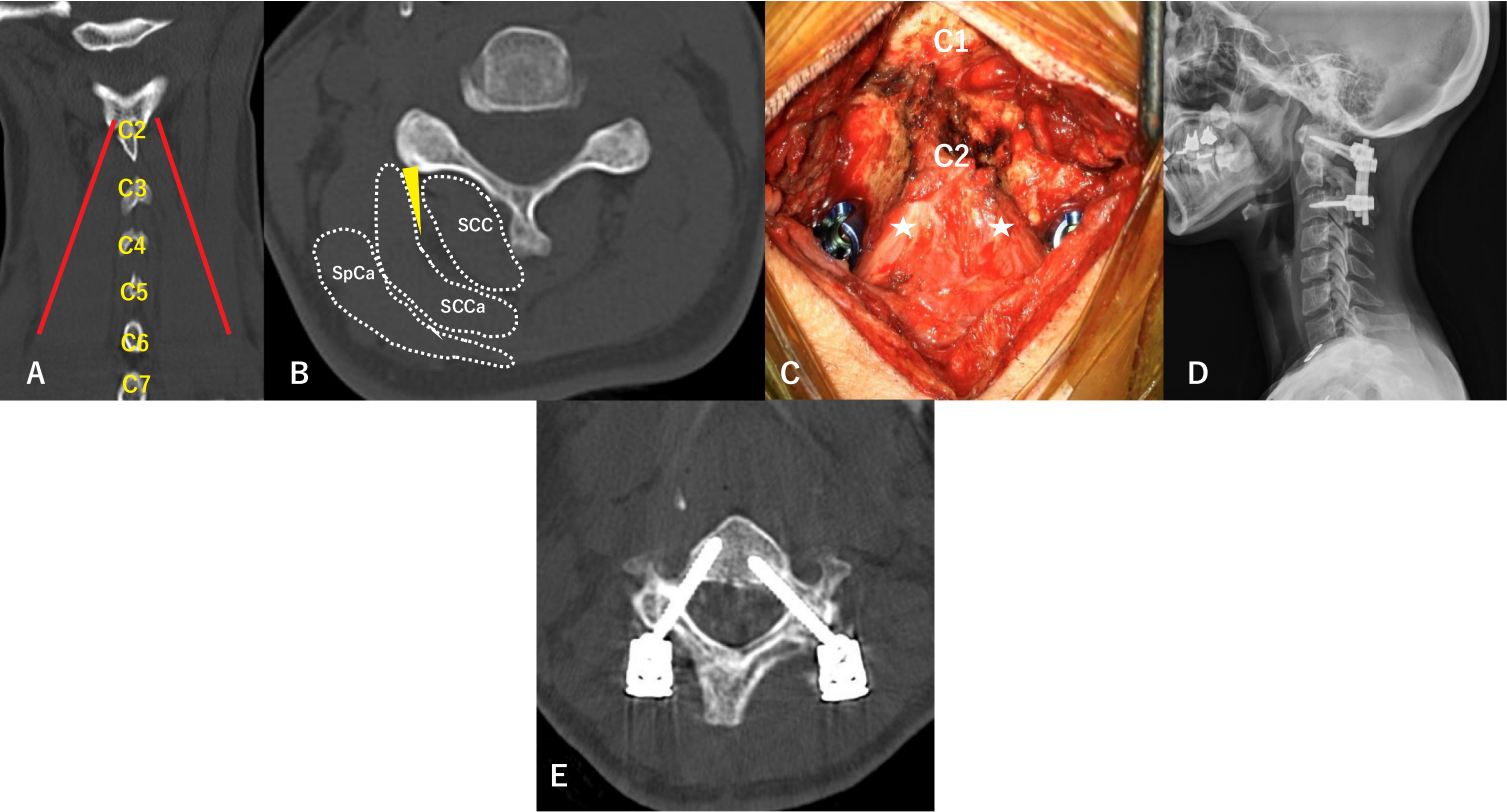
Preoperative, intraoperative, and Postoperative findings during posterior C1–3 fixation. (A) Preoperative CT demonstrating the anatomical course of the semispinalis cervicis (SSC) muscle (red line). (B) Preoperative CT demonstrating the anatomical structure of the posterior cervical muscle and fat plane (yellow area) at the C3 level. SSC, Semispinalis cervicis muscle; SCCa, semispinalis capitis; SpCa, splenius capitis. (C) Intraoperative photograph showing the preserved SSC (★) during screw placement; screws placed via a lateral trajectory beneath SSC retraction. (D) Postoperative X-ray demonstrating appropriate alignment and secure placement of the obliquely inserted cervical pedicle screws. (E) Postoperative CT demonstrating that pedicle screws were inserted nicely and obliquely.

Under general anesthesia, a midline posterior cervical incision was made, and the posterior elements of C1–C3 were exposed. The SSC muscle was identified based on its anatomic course, originating from the T1–T6 transverse processes and inserting onto the C2 spinous process (Fig. 2A). The rectus capitis posterior major and obliquus capitis inferior muscles were detached from the C2 spinous process, while the SSC was preserved. Selective SSC preservation allowed adequate exposure of the C2–C3 facet joints while minimizing muscle trauma. During this process, the entry points for the C1 and C3 screws were easily visualized by minimally retracting the SSC medially using a periosteal elevator. Lateral mass screws (3.5 mm in diameter, 26 mm in length for both sides) were inserted at C1, and pedicle screws (3.5 mm in diameter, 24 mm in length for both sides) were placed at C3 using intraoperative three-dimensional cone-beam CT navigation to ensure optimal trajectory and positioning (Fig. 2B and C). The total operative time was 171 minutes, with an estimated blood loss of 5 mL.

**Figure 3.**
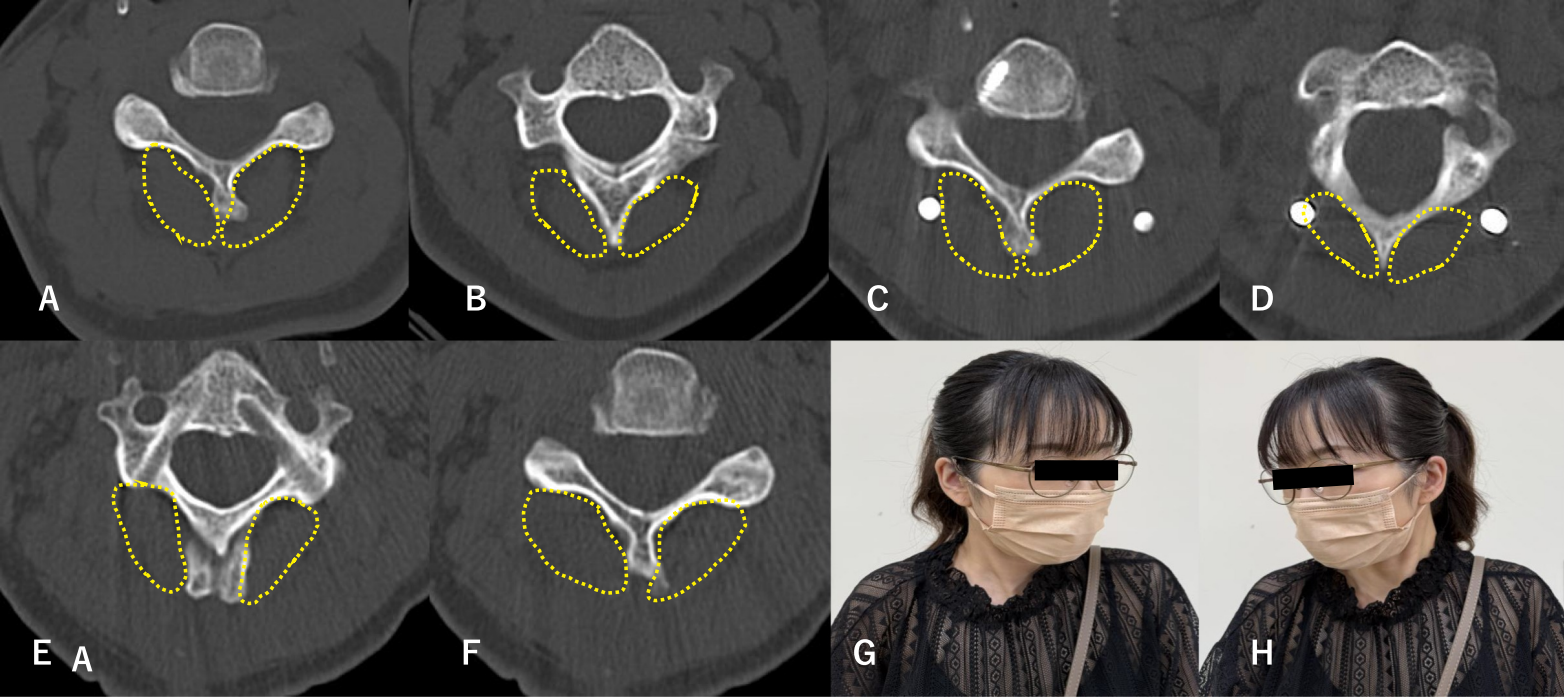
CT images obtained at the C2 and C3 vertebral levels and Clinical photographs of the patient. (A and B) Preoperative axial CT images demonstrating SSC muscle bulk at both C2 and C3 levels. (C and D) Postoperative axial CT images showing that the SSC muscle has been preserved intact at the corresponding levels after fixation surgery. (E and F) Axial postoperative CT images demonstrating intact SSC muscle (yellow line) at C2 and C3 after hardware removal. (G and H) Clinical photographs of the patient performing head rotation, demonstrating recovery of motion without restriction.

Preoperative and postoperative CT images confirmed that the SSC muscle was preserved throughout the procedure (Fig. 3). The postoperative course was uneventful, and the patient was discharged on postoperative day 7 without neurological deficits. At the 1-year follow-up, CT demonstrated near-complete fusion of the pars interarticularis (Fig. 1E and F). However, the patient reported persistent limitations in cervical rotation, with a total range of motion (ROM) of approximately 60°. Furthermore, she experienced severe pain during rotational movement, rated as 8 on the Numerical Rating Scale (NRS). Given these findings, implant removal was performed. The original surgical plane was re-entered, and the SSC remained intact with minimal adhesions. Implant removal was straightforward. Follow-up CT showed preserved SSC at both C2 and C3 levels. Following the removal, the patient exhibited marked improvement in cervical rotation, with the total ROM increasing to approximately 140°. Additionally, the rotation-induced pain completely resolved (NRS 0), and she remained symptom-free (Fig. 3).

## Discussion

C2 Hangman’s fractures typically result from a hyperextension injury. Although Levine–Edwards type I fractures are usually managed conservatively, fractures with instability, large fracture gaps, or failure to achieve union with external immobilization require surgical fixation.

Posterior fixation spanning the atlanto-axial complex (C1–C3) provides strong biomechanical stability and is widely used for unstable Hangman’s fractures. However, fusion constructs that include C1 significantly restrict cervical rotational motion – up to 70% in some reports. Although implant removal after bony consolidation may help restore motion, conventional posterior approaches often cause substantial paraspinal muscle injury, which has been associated with chronic axial neck pain and progressive cervical kyphosis.

The SSC plays an essential role in cervical extension, rotation, and posterior tension band stability. Injury to the SSC has been associated with postoperative kyphosis and axial symptoms. Previous literature on cervical laminoplasty has demonstrated that preserving the SSC helps maintain postoperative alignment. However, preserving the SSC during C1–C3 fixation is technically challenging because of its firm attachment to the C2 spinous process. Identifying the fat plane between the SSC and SCCa, carefully dissecting between these muscles, and determining an appropriate pedicle screw entry point are essential for minimizing muscle trauma (Fig. 2B).

Although the muscle-preserving technique may increase the technical complexity of the procedure, its potential benefits likely extend beyond Hangman’s fracture management.^[^8–11^]^ This technique may be applicable to other posterior cervical fusion procedures – including occipitocervical constructs – to help preserve rotational mobility, reduce muscle-related axial pain, and facilitate easier implant removal.

Regarding surgical efficiency, the operative time in the present case was 171 minutes, with a total blood loss of 5 ml. This operative time was comparable to that reported in previous studies and was considered within an acceptable range, even including the time required for the navigation setup and the meticulous procedure for selective SSC preservation.

However, the limitations of this report include its single-case nature, the absence of quantitative motion analysis, and the relatively short follow-up period after implant removal. Larger studies with standardized functional assessments and long-term outcomes are required to clarify the true clinical benefits of SSC preservation.

## Conclusion

Posterior cervical fixation with preservation of the SSC muscle is a feasible and technically sound strategy for unstable C2 Hangman’s fractures. SSC preservation facilitated uncomplicated implant removal and may help maintain postoperative cervical rotational function and alignment stability. Further accumulation of cases with long-term functional evaluation is warranted to validate these findings.

## Ethical approval

Ethical approval was waived by the institutional review board because this is a single case report. Written informed consent was obtained from the patient for publication of this case report and accompanying images.

## Data Availability

All data relevant to the case are included within the article.
